# Effects of Heat-Killed *Lactobacillus acidophilus* IDCC 3302 on Skin Aging Parameters: A Randomized, Double-Blind, Placebo-Controlled Trial in Healthy Adults

**DOI:** 10.3390/nu18040596

**Published:** 2026-02-11

**Authors:** Hayoung Kim, Won Yeong Bang, Kyu Ho Jeong, Young Hoon Jung, Jungwoo Yang, Jin Seok Moon

**Affiliations:** 1 ILDONG Bioscience Co., Ltd., Pyeongtaek-si 17957, Gyeonggi-do, Republic of Korea; young@ildong.com (H.K.); yeong0417@ildong.com (W.Y.B.); 2 ILDONG Pharmaceutical Co. Ltd., Seoul 06752, Republic of Korea; whoai@ildong.com; 3 School of Food Science and Biotechnology, Food and Bio-Industry Institute, Kyungpook National University, Daegu 41566, Gyeongsang-do, Republic of Korea; younghoonjung@knu.ac.kr; 4 Department of Microbiology, College of Medicine, Dongguk University, Gyeongju 38066, Gyeongsang-do, Republic of Korea; dbl3jwy@dongguk.ac.kr

**Keywords:** postbiotic, *Lactobacillus acidophilus* IDCC 3302, skin aging, elasticity, hydration, transepidermal water loss

## Abstract

Background/Objectives: Skin aging is a multifactorial process driven by intrinsic and extrinsic factors. Orally administered postbiotics may support skin homeostasis; however, clinical validation of their efficacy remains limited. In this study, we aimed to examine the effect of oral supplementation with the heat-killed lactic acid bacterium *Lactobacillus acidophilus* IDCC 3302 (ID-ACT 3302) on skin aging-related markers in healthy adults with periorbital wrinkles. Methods: In this 12-week, randomized, double-blind, placebo-controlled trial, 100 participants were assigned to receive either ID-ACT 3302 or a placebo. The prespecified primary endpoint was the change in crow’s-feet wrinkles at week 12, assessed using investigator visual wrinkle grading and PRIMOS^lite^. Secondary endpoints included skin hydration, transepidermal water loss (TEWL), skin elasticity indices (including the overall elasticity index R2), and global improvement scores. Results: In the full analysis set, wrinkle grade, hydration, TEWL, and elasticity improved over 12 weeks in both groups; however, ID-ACT 3302 exhibited no superiority over placebo for the primary wrinkle endpoints. The overall elasticity index R2 exhibited significant inter-group difference, whereas R5, R7, hydration, TEWL, and global assessment scores improved consistently in both groups. In the absence of multiplicity adjustments for secondary endpoints, the R2 finding is considered exploratory. Safety outcomes were comparable between the groups, with no serious adverse events. Conclusions: The 12-week supplementation with ID-ACT 3302 was safe and well tolerated. However, no placebo-adjusted benefit was observed for the prespecified primary wrinkle endpoints. An exploratory between-group difference was observed for the Cutometer overall elasticity index (R2), but because secondary endpoints were not multiplicity-adjusted and the effect size was small, this finding should be interpreted as hypothesis-generating and requires confirmation in adequately powered trials.

## 1. Introduction

Skin aging is a gradual, multifactorial process regulated by intrinsic and extrinsic factors. Intrinsic aging is characterized by reduced cellular turnover, diminished dermal matrix synthesis, and impaired antioxidant defence, which concomitantly induce dermal thinning, loss of elasticity, and wrinkle formation. Extrinsic factors, particularly chronic ultraviolet (UV) exposure, environmental pollutants, and oxidative stress, accelerate collagen and elastin degradation, increase transepidermal water loss (TEWL), and promote chronic low-grade inflammation, resulting in photoaging [1,2,3]. Clinical manifestations of these changes include fine lines, sagging, and texture alterations, which are associated with compromised barrier integrity and impaired hydration [4,5].

In addition to topical treatments, growing interest in “ingestible skincare” approaches is evident, which focus on improving dermal health through endogenous processes. In this context, probiotics and related microbial preparations have attracted significant research attention. In addition to live microorganisms, non-viable microbial cells and cell components, described as paraprobiotics or postbiotics, have been explored, which can retain biological activity despite heat inactivation, and provide advantages in formulation stability and safety [6,7]. In the present study, we use the term “postbiotic” to refer to a preparation of heat-killed *Lactobacillus acidophilus* IDCC 3302, considering that related concepts, such as paraprobiotics, denote non-viable microorganisms with documented biological effects [8]. Increasing evidence has shown that heat-killed bacterial strains exert antioxidative, anti-inflammatory, and barrier-supportive effects associated with skin homeostasis [9,10,11].

*L. acidophilus* IDCC 3302 (commercially known as ID-ACT 3302) is a heat-killed lactic acid bacterium originally isolated from healthy Korean infants. Preclinical studies have revealed the protective effects of ID-ACT 3302 against UVB-induced photodamage. In human keratinocytes, tyndallized *L. acidophilus* IDCC 3302 reduced UVB-induced cytotoxicity and oxidative stress, downregulated pro-inflammatory cytokines (IL-1β, TNF-α, and IL-8), suppressed mitogen-activated protein kinase activation, and decreased the expression of matrix metalloproteinases (MMP-1, -2, and -9), the central mediators of collagen degradation [12,13]. Simultaneously, it enhanced the expression of barrier- and hydration-related markers, such as filaggrin, involucrin, and transforming growth factor-β (TGF-β), indicating its multifaceted protective role in photoaging.

Consistently, the findings of animal experiments reflected that oral administration of heat-killed *L. acidophilus* IDCC 3302 in murine models improved skin hydration, normalized epidermal thickness, and attenuated UV-induced wrinkle formation, which were in line with its systemic immunomodulatory and antioxidative actions [14]. Collectively, these preclinical data indicate that ID-ACT 3302 is a promising candidate for improving skin health, particularly as a postbiotic ingredient in oral functional products, such as dietary supplements.

Despite mechanistic and preclinical evidence, clinical validation of the potential of ID-ACT 3302 in humans remains limited. Only a small number of randomized controlled trials have analyzed oral paraprobiotics or postbiotics, focusing on objective dermatological endpoints such as elasticity, hydration, and wrinkle depth [9,15]. Many of these studies reported modest effects over 8–12-week periods and highlighted the necessity of further trials using standardized instrument-based assessments [14,16,17,18,19].

Therefore, in this study, we aimed to evaluate the clinical efficacy and safety of oral supplementation with heat-killed *L. acidophilus* IDCC 3302 in adults exhibiting mild-to-moderate photoaging. In this 12-week, randomized, double-blind, placebo-controlled, parallel-group study, we assessed the effects of ID-ACT 3302 on periorbital wrinkle parameters, considered the prespecified primary endpoint, as well as on skin hydration, TEWL, and elasticity indices (including R2 as a key secondary endpoint). Wrinkles were evaluated using visual grading and three-dimensional optical imaging, and skin barrier and mechanical properties were assessed using standardized instrument-based measurements of hydration, transepidermal water loss (TEWL), and elasticity. This study can potentially generate robust clinical data on the dermatological profile of this postbiotic strain, while clearly distinguishing primary from secondary outcomes.

## 2. Materials and Methods

### 2.1. Study Design and Participants

The study was conducted following the Declaration of Helsinki and the Korean Good Clinical Practice guidelines. The protocol followed in this randomized, double-blind, placebo-controlled clinical trial and the informed consent forms were reviewed and approved by the Institutional Review Board of Chung-Ang University Hospital, Seoul, Republic of Korea (approval date: 20 February 2017; protocol version 1.0; IRB No. 1712-003-268); moreover, this study was registered at the Clinical Research Information Service (CRIS, KCT0002863). All the participants provided written informed consent before participating in the assessment. The study was conducted between April 2017 and November 2017 at Chung-Ang University Hospital. Screening began on 18 April 2017, and the last participant completed the final visit on 13 November 2017.

Men and women (aged 29–59 years) with visible facial wrinkles, who were visiting a dermatology outpatient clinic, were recruited for this study. At screening (visit 1), eligibility was assessed based on medical history, physical examination, dermatological evaluation, and routine laboratory testing, with two major inclusion criteria: (i) age between 29 and 59 years, and (ii) presence of periorbital wrinkles with an investigator-assessed visual wrinkle grade ≥3 (0–9 scale) based on the MFDS guideline [20].

To minimize confounding factors and ensure participant safety, individuals with active or chronic skin diseases, such as atopic dermatitis or psoriasis, or with marked lesions (e.g., prominent moles, acne, erythema, and telangiectasia) in the evaluation area were excluded. Participants who had undergone the following facial procedures that potentially affect skin conditions were ineligible: (1) chemical peeling or other intensive facial treatments within 1 month, or (2) botulinum toxin or filler injections within 6 months. The use of topical steroid formulae on the face, oral retinoids, or systemic steroid administration within 3 months before baseline, and the use of wrinkle-improving functional cosmetics containing retinoids, retinol, or alpha-hydroxy acids, as well as intensely moisturising products within 2 weeks, was not permitted.

Throughout the study, participants were instructed to maintain their usual home skincare and makeup routines as much as possible. To minimize variability in topical skincare, the same moisturizer was provided to both groups at baseline (visit 2) and week 6 (visit 3). During the intervention period, the use of anti-wrinkle functional cosmetics (e.g., retinoids/retinol/alpha-hydroxy acids) and highly moisturizing/elasticity-targeting products was prohibited. Any changes in cosmetic use (including makeup removers and topical products such as creams, serums, and masks) were recorded in a cosmetic-use diary and reviewed at each follow-up visit.

Concomitant medications and supplements associated with potential impacts on dermatologic parameters or metabolic status were restricted. Moreover, the exclusion criteria included the use of anti-obesity medications (including lipase inhibitors, antidepressants, or appetite suppressants), oral contraceptives or other hormonal agents, or diuretics within 1 month before study initiation. Intake of probiotic functional foods or consumption of fermented milk products ≥4 times weekly within 1 week before screening, as well as use of antioxidant, hyaluronic acid- or collagen-containing supplements, evening primrose oil or vitamin A-, C- or E-containing drugs or health functional foods within 2 weeks before screening, was considered for exclusion. Participants who had received any other investigational drugs or foods within one month before baseline or who planned to participate in another clinical trial during the study period were not enrolled.

Participants with clinically significant abnormalities revealed through routine laboratory tests were excluded; the abnormal parameters included aspartate aminotransferase (AST), alanine aminotransferase (ALT), or γ-glutamyl transpeptidase (γ-GTP) levels ≥3-fold the upper limit of normal (ULN) at the study site, serum creatinine ≥2-fold ULN, or thyroid-stimulating hormone (TSH) ≤0.1 or ≥10 μIU/mL. Additional exclusion criteria were uncontrolled hypertension (blood pressure ≥160/100 mmHg after at least 10 min of rest) or diabetes mellitus (fasting blood glucose ≥180 mg/dL), a history of or current schizophrenia, depression or drug dependence, pregnancy or lactation or planned pregnancy within 3 months, a history of hospitalisation and pharmacologic or rehabilitative treatment for alcohol consumption or alcohol-induced disorders, heart disease or central nervous system disorders, current smoking or smoking cessation within 1 year before screening, and any other condition ruled by the investigator to make study participation inappropriate.

### 2.2. Sample Size Calculation

The sample size was determined, considering the aim to detect inter-group differences in wrinkle improvement after 12 weeks of supplementation. The null hypothesis indicated that the mean change in the primary wrinkle parameter would be equal in the ID-ACT 3302 and placebo groups (H_0_: μ_t_ = μc), and the alternative hypothesis was that the means would differ (H_1_: μ_t_ ≠ μc). Based on a previous randomized trial that evaluated a similar wrinkle endpoint [21], in which the mean change was −0.055 and 0.033 in the active and placebo groups, respectively (estimated pooled standard deviation [SD] of 0.139). Assuming a two-sided significance level of 5% (α = 0.05), a type II error rate of 20% (*β* = 0.20; 80% power), and equal allocation between groups, the required sample size was calculated using the following formula:

$$n=\frac{{2\left(Z_{\frac{\alpha }{2}}+Z_{\beta }\right)}^{2}\sigma^{2}}{{∆}^{2}}$$

where *Z_α_*_/2_ and *Z_β_* are the standard normal critical values for *α*/2 and *β*, respectively, *σ* is the SD (0.139) and *Δ* is the expected mean difference between groups (−0.088). Substituting these values yielded approximately 40 participants per group. In preparation for an anticipated dropout rate of 20%, the target enrollment was set as 50 participants per group (total *n =* 100), with at least 40 participants per group planned for inclusion in the per-protocol efficacy analysis.

### 2.3. Study Products and Intervention

The experimental product, ID-ACT 3302, comprised tyndallized *L. acidophilus* IDCC 3302 in capsule form. Participants allocated to the test group received one capsule once daily with water for 12 weeks; each capsule contained 1.0 × 10^10^ cells of heat-killed (tyndallized) *L. acidophilus* IDCC 3302 (daily intake: 1.0 × 10^10^ cells). The placebo consisted of a visually indistinguishable capsule without the active bacterial component. Each placebo capsule contained corn starch (97.3%), gardenia yellow color powder (1.0%), Caramel color powder (0.5%), cochineal extract color (0.2%), and magnesium stearate (1.0%), with a total fill weight of 350 mg. The placebo was administered at a dose of one capsule once daily with water for 12 weeks, following the same schedule and instructions as the test product.

After confirmation of eligibility and collection of informed consent, the participants were enrolled and randomly assigned, in the order of registration, to receive either ID-ACT 3302 or the placebo. Both the participants and investigators remained blinded to the treatment allocation throughout the trial. During the intervention period, participants were asked to maintain their usual lifestyle and skincare routines in addition to the specific restrictions outlined in the eligibility criteria.

**Figure 1:**
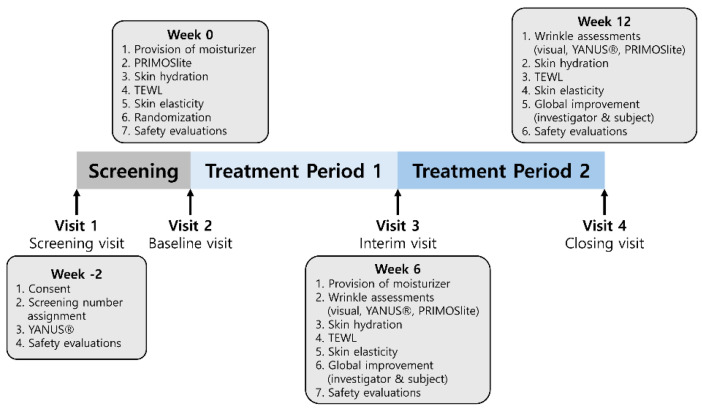
Study design and the visits and assessment schedule. After a screening period (week −2–0, visit 1), eligible participants were randomized at baseline (week 0, visit 2) to receive ID-ACT 3302 or placebo once daily for 12 weeks. Follow-up visits were conducted at week 6 (visit 3) and week 12 (visit 4). TEWL, transepidermal water loss. At screening, investigator visual wrinkle grading and safety evaluations were performed. At baseline, PRIMOS^lite^ wrinkle imaging, skin hydration, transepidermal water loss (TEWL), skin elasticity, and safety were assessed, and moisturizer was provided. At weeks 6 and 12, wrinkle assessments (visual and PRIMOS^lite^), skin hydration, TEWL, skin elasticity, global improvement (investigator and participant), and safety evaluations were repeated according to the schedule. To minimize variability in skincare during the intervention, a standard moisturizer was provided to all randomized participants at baseline (visit 2) and again at week 6 (visit 3). Participants in both groups were instructed to use the same provided moisturizer during the study period. In addition, participants recorded any changes in cosmetic products in a cosmetic use diary, which was reviewed by study staff at subsequent visits.

The study comprised four scheduled visits: screening (visit 1), baseline assessment and randomization (visit 2, day 0), week 6 (visit 3, days 42 ± 7), and week 12 (visit 4, days 84 ± 5). The timing of visits and schedule of efficacy and safety assessments are schematically summarized in Figure 1.

### 2.4. Efficacy Assessments

The efficacy endpoints were evaluated using a combination of clinical grading scales, instrumental measurements, and global improvement ratings associated with wrinkles and skin barrier function.

Periorbital wrinkle severity was assessed by investigator visual wrinkle grading using a 10-point ordinal scale (0–9) in accordance with the guideline for Efficacy Evaluation of Functional Cosmetics II (Anti-wrinkle functional cosmetics) issued by the Ministry of Food and Drug Safety (MFDS), Republic of Korea [20]. Standardized facial photographs were captured using the YANUS facial imaging system under predefined illumination conditions (standard visible light, polarized light, and UV light) and were used as supportive documentation for the investigator’s visual assessment. Instrumental wrinkle assessments were additionally performed using PRIMOS^lite^ (GFMesstechnik GmbH, Teltow, Germany), a non-contact optical three-dimensional in vivo measurement system, and the PRIMOS^lite^-based parameters included surface roughness indices and eye-wrinkle volume at the left and right crow’s feet.

To evaluate barrier function and mechanical properties, skin hydration, TEWL, and skin elasticity were measured at predefined facial and forearm sites on the left and right sides at baseline, week 6, and week 12 [22,23,24]. Skin hydration was measured using a Corneometer^®^ CM825 (Courage&Khazaka electronic GmbH, Cologne, Germany), TEWL using a Tewameter^®^ TM 300, and skin elasticity indices R2 (overall elasticity, Ua/Uf), R5 (immediate elasticity, Ur/Ue), and R7 (elasticity ratio, Ur/Uf) using a Cutometer^®^ MPA580 (Courage&Khazaka electronic GmbH, Cologne, Germany). Skin hydration values are expressed in arbitrary units (AU), TEWL in g/h/m^2^, PRIMOS^lite^ roughness parameters in µm and wrinkle volume in mm^3^, and Cutometer indices (R2, R5, and R7) are dimensionless ratios. For each parameter, changes were calculated compared with the baseline values, separately for the left and right sides, as well as for the means of both sides.

Perceived improvement was evaluated by both the investigator and participants. At weeks 6 and 12, both the investigator and participants rated global improvement using a 5-point ordinal scale (1 = markedly improved, 2 = improved, 3 = no change, 4 = worsened, and 5 = markedly worsened) compared with baseline. At weeks 6 and 12, the investigators rated global improvement using a predefined 5-point scale, and the participants provided self-assessment scores using a similar structured scale.

The primary efficacy endpoint was the change in wrinkle parameters at week 12 compared with baseline, which was assessed using investigator visual wrinkle grading and PRIMOS^lite^ measurements at the periorbital region. Secondary endpoints included changes in wrinkle measures at week 6; changes in skin hydration, TEWL, and elasticity at weeks 6 and 12; and investigator- and participant-rated global improvement scores at weeks 6 and 12.

### 2.5. Safety Assessments

The safety was monitored throughout the 12-week intervention period. At each visit, the participants were interviewed for reports of adverse events (AEs), and all reported AEs were documented and coded according to the MedDRA (ver. 20.1) ‘preferred terms’. For each treatment group, the proportion of participants who experienced at least one AE and the total number of AEs were calculated.

Clinical laboratory tests, including haematology, serum biochemistry, and urinalysis, were conducted at screening (visit 1) and at week 12 (visit 4). The selected urinalysis variables were categorised as normal or abnormal. Vital signs (pulse rate and blood pressure) and body weight were measured during the same visit. Body weight was measured using a calibrated digital scale with participants wearing light clothing and no shoes. Measurements were obtained at the time of the participant’s clinic visit (routine clinic hours) rather than at a fixed time of day.

### 2.6. Analysis of Populations and Statistical Methods

Three analysis populations were defined: the safety set included all the randomized participants who ingested at least one dose of the study product (*n =* 100; ID-ACT 3302, *n =* 50; placebo, *n =* 50). The full analysis (FA) set comprised participants who met the predefined criteria for inclusion in the efficacy analyses and closely approximated the intention-to-treat population (*n =* 92; ID-ACT 3302, *n =* 45; placebo, *n =* 47). The per-protocol (PP) set included participants who completed the trial without major deviations in the protocol (*n =* 89; ID-ACT 3302, *n =* 43; placebo, *n =* 46). The PP set served as the primary population for efficacy evaluations, and the FA set was additionally analyzed for sensitivity purposes.

All statistical analyses were performed using SAS^®^ software (version 9.4; SAS Institute, Cary, NC, USA). For the primary endpoint, intra-group changes in wrinkle parameters assessed at week 12 compared with baseline were analyzed using paired *t*-tests. Inter-group differences in changes at each time point were evaluated using analysis of covariance (ANCOVA) with baseline values as covariates and two-sample *t*-tests. When the assumption of normality was not met, the Wilcoxon rank-sum test was used as a non-parametric alternative.

For secondary continuous endpoints (wrinkle measurements at week 6; skin hydration, TEWL, and elasticity at weeks 6 and 12), intra-group changes from baseline were analyzed using paired *t*-tests; inter-group differences were assessed using ANCOVA and two-sample *t*-tests, with Wilcoxon rank-sum tests applied when appropriate. Considering the number of secondary endpoints and time points examined, no formal adjustment for multiple comparisons was performed. Accordingly, the analyses of the secondary endpoints were considered exploratory, and the *p*-values for these outcomes should be interpreted with caution in the context of multiplicity. Investigator and participant global assessment scores were compared between the groups using either two-sample *t*-tests or Wilcoxon rank-sum tests, according to the distribution of the data.

For safety analyses, the proportion of participants with at least one AE was compared between the groups using chi-square or Fisher’s exact tests, as appropriate. For continuous laboratory parameters, intra-group changes were evaluated using paired *t*-tests, and inter-group differences in changes were analyzed using two-sample *t*-tests. Intra-group changes were assessed using McNemar’s test for urinalysis parameters that were classified as normal or abnormal. Vital signs and body weight were analyzed using paired *t*-tests for intra-group changes from baseline and two-sample *t*-tests for inter-group comparisons.

## 3. Results

### 3.1. Subject Disposition and Analysis Sets

**Figure 2:**
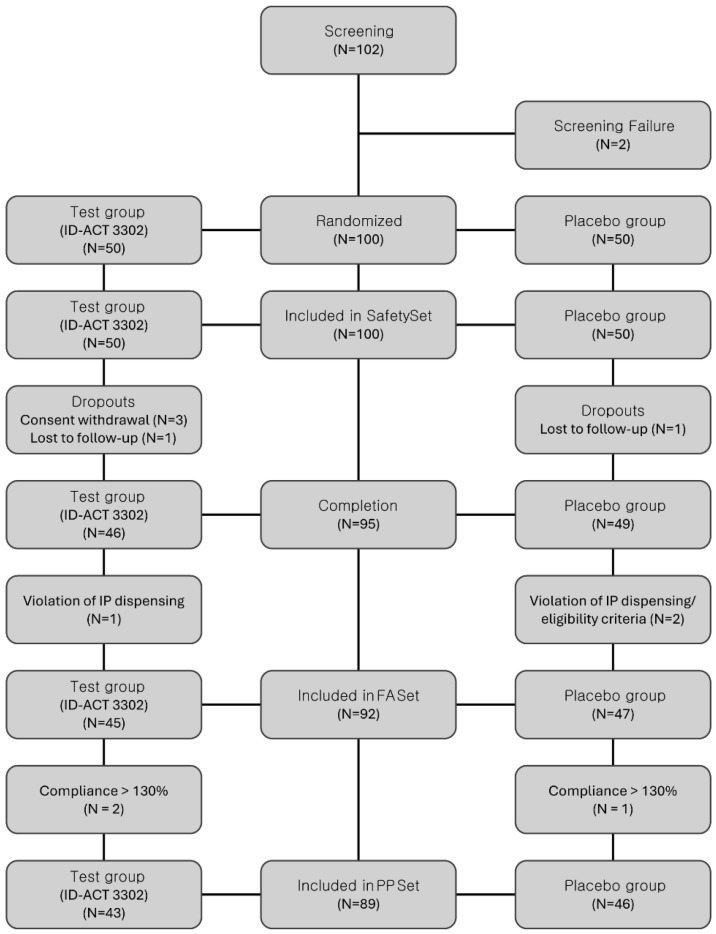
CONSORT flow diagram of participant disposition throughout the study. A total of 100 participants were randomly assigned to the ID-ACT 3302 and placebo groups (1:1). The figure summarizes the number of participants screened, randomized, allocated to each group, lost to follow-up or excluded, and included in the Full Analysis and Per-Protocol sets. PP set, participants without major protocol deviations and with compliance within the prespecified range. IP, investigational product.

One hundred participants were randomized in a 1:1 ratio to receive ID-ACT 3302 (*n =* 50) or placebo (*n =* 50). The flow of participants through screening, randomization, follow-up, and inclusion in the analyses is presented in Figure 2.

The PP set (ID-ACT 3302, *n =* 43; placebo, *n =* 46) served as the primary population for the efficacy analyses, and the FA set (ID-ACT 3302, *n =* 45; placebo, *n =* 47) was considered for sensitivity analyses conducted to assess the robustness of the findings. The baseline demographic and clinical characteristics, including lifestyle information, were comparable between the two groups (Table 1). The reasons for exclusion from the FA and PP sets, including withdrawal of consent, loss to follow-up, and major protocol deviations, are shown in Figure 2.

### 3.2. Primary Endpoint: Wrinkle Improvement at Week 12

The change in wrinkle parameters as assessed at week 12 using investigator visual wrinkle grading and PRIMOS^lite^ was the primary efficacy endpoint.

**Figure 3:**
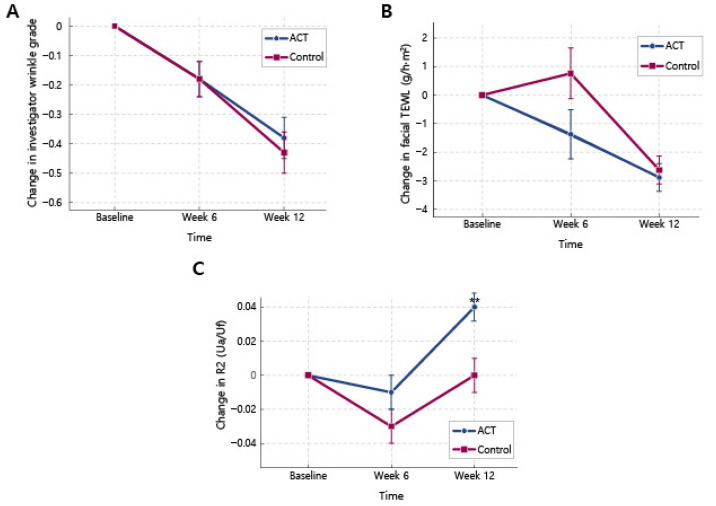
Time course of key skin parameters in the ID-ACT 3302 and placebo groups (PP set). ACT indicates the ID-ACT 3302 group and Control indicates placebo (PP set: ID-ACT 3302, *n =* 43; placebo, *n =* 46). Data are changes from baseline (baseline = 0); lower wrinkle grade/TEWL indicates improvement, and higher R2 indicates increased elasticity. TEWL, transepidermal water loss; SEM, standard error of the mean. (**A**) Variation in the visual wrinkle grade at the crow’s feet assessed using the investigator visual wrinkle grades (mean of left and right sides). (**B**) Variation in facial transepidermal water loss (TEWL) at the abovementioned sites measured using the Tewameter^®^. (**C**) Variation in overall skin elasticity index R2 (Ua/Uf) at the periorbital/nasal-tip sites measured using the Cutometer^®^. Values represent mean ± SEM. Intra-group changes from baseline were analyzed using paired *t*-tests. Inter-group variations in the change from baseline values were analyzed using ANCOVA with baseline values as covariates. When appropriate, the Wilcoxon rank-sum test was used. ** *p* < 0.01 vs. baseline (intra-group).

In the FA set, visual wrinkle grades at the crow’s feet decreased significantly from the baseline in both groups; at the left crow’s feet, mean wrinkle grades decreased in both the ID-ACT 3302 and placebo groups, and similar reductions were observed on the right side and for the mean of both sides (Table 2). At week 12, no inter-group comparisons revealed statistically significant changes in the investigator visual wrinkle grades. Consistent results were recorded in the PP and FA sets. The gradual changes in mean investigator visual wrinkle grades (combined left and right values) are shown in Figure 3A.

The PRIMOS^lite^-assisted measurements revealed small changes in the surface roughness indices (Ra and Rz) and eye-wrinkle volume in both the left and right crow’s feet over 12 weeks. Significant intra-group changes included gradual improvement in wrinkles during the study period; however, none of the PRIMOS^lite^ parameters differed significantly between the ID-ACT 3302 and placebo groups at week 12 (Table 2). Analyses based on the mean values of the left and right crow’s feet led to similar conclusions.

Overall, in this 12-week trial, clinical wrinkle grades exhibited significant intra-group improvements in ID-ACT 3302 and placebo groups; however, ID-ACT 3302 did not exhibit superiority over placebo in the prespecified primary wrinkle endpoints.

### 3.3. Secondary Wrinkle Outcomes at Week 6

Secondary analyses of wrinkles assessed at week 6 using investigator visual wrinkle grading and PRIMOS^lite^ revealed early improvements from baseline in both groups (Table 3); at week 12, no statistically or clinically significant differences were observed in wrinkle outcomes. These findings revealed that the magnitude and trajectory of wrinkle improvement were largely comparable between the ID-ACT 3302 and placebo groups over 12 weeks.

### 3.4. Skin Hydration and Transepidermal Water Loss

At week 12, Corneometer^®^ CM825-based analysis of skin hydration revealed a significant increase from baseline at facial sites located at the intersection of the crow’s feet area and the nasal tip in both the ID-ACT 3302 and placebo groups. Similar improvements were observed at the volar forearm (Table 4). The mean values for the left and right facial and forearm sites reflected significant increases in skin hydration in both groups compared with the baseline counterparts. No significant inter-group differences were detected at any site or time point. The FA analyses supported these findings.

TEWL, assessed using the Tewameter^®^ TM 300, decreased significantly from baseline at facial sites in both groups, indicating gradual improvement in skin barrier function (Table 4). At week 12, comparable reductions in facial TEWL (left, right, and mean values) were detected in the ID-ACT 3302 and placebo groups, with no significant inter-group differences. Overall, TEWL slightly changed at the forearm sites, with a significant increase at week 6 in the ID-ACT 3302 group; however, no consistent or clinically significant inter-group differences were evident. The time course of the changes in mean facial TEWL is shown in Figure 3B.

These results indicate that both ID-ACT 3302 and placebo were associated with improvements in skin hydration and reductions in facial TEWL over the 12 weeks, and that an additional benefit of ID-ACT 3302 over placebo could not be determined for these parameters.

### 3.5. Skin Elasticity and Global Improvement

At the crow’s feet/nasal-tip sites, the overall elasticity index R2 (Ua/Uf; higher values indicating better overall elasticity) showed minor but statistically significant inter-group differences in favour of ID-ACT 3302 among the secondary endpoints.

In the FA set, the mean R2 (averaged across the left and right facial sites) increased modestly from the baseline in both groups (Table 5); however, the improvement was significantly greater in the ID-ACT 3302 group than in the placebo group (*p* < 0.05). The corresponding 95% confidence interval for the inter-group difference did not exceed zero (Table 5), which was consistent with a modest treatment effect. The time course of the changes in the mean R2 is presented in Figure 3C, where the ID-ACT 3302 curve remains above that of the placebo curve at week 12. Similar changes revealed by the analysis of the PP set supported these findings.

In contrast, the immediate elasticity parameter R5 (Ur/Ue) and elasticity ratio R7 (Ur/Uf) increased significantly from baseline in both groups, reflecting an overall improvement in skin elasticity over time; however, no significant inter-group differences were observed at week 12 (Table 5).

Moreover, global improvement was evaluated using investigator- and participant-rated scores. At week 12, the mean investigator global assessment scores were comparable in the ID-ACT 3302 and placebo groups; the self-assessment scores, estimated by the participants, showed a comparable pattern, thereby indicating no clear advantage of ID-ACT 3302 over placebo in terms of perceived overall improvement (Table 5).

Overall, these findings indicate that ID-ACT 3302 supplementation was associated with a modest but statistically significant improvement in the global elasticity index R2 compared with the placebo, whereas other elasticity indices (R5 and R7) and global improvement scores improved to a similar extent in both groups. Considering the number of secondary endpoints evaluated and the absence of multiplicity adjustment, the R2 result should be regarded as exploratory and hypothesis-generating instead of as definitive evidence of a large cosmetic effect.

### 3.6. Safety

Safety analyses were conducted based on the safety set, which included all 100 randomized participants who ingested ID-ACT 3302 or a placebo at least once (ID-ACT 3302, *n =* 50; placebo, *n =* 50). During the 12-week intervention, AEs were reported by one participant (one event) in the ID-ACT 3302 group and three participants (four events) in the placebo group. The incidence of AEs did not significantly differ between the groups (*p* = 0.6173), and all events reflected mild intensity. No serious AEs occurred, and none of the participants discontinued the study owing to AEs. None of the AEs were judged to be related to the study products in either group (Table 6). Haematological and clinical chemistry parameters exhibited no statistically significant inter-group differences after 12 weeks of ingestion (Table 6). The pulse rate, systolic and diastolic blood pressures, and body weight remained stable throughout the study, with no significant inter-group differences compared with the baseline (Table 6). Overall, these data indicate that the 12-week ingestion of ID-ACT 3302 is safe and well tolerated in this population of healthy adults with mild-to-moderate photoaging.

## 4. Discussion

This randomized, double-blind, placebo-controlled trial showed the potential dermatological benefits of oral supplementation with heat-killed lactic acid bacterium *L. acidophilus* IDCC 3302 (ID-ACT 3302) over a 12-week study period in adults with mild-to-moderate photoaging. Investigator visual wrinkle grading and PRIMOS^lite^-based assessments revealed no significant superiority of ID-ACT 3302 over placebo in terms of the primary endpoints, such as changes in periorbital wrinkle parameters. Therefore, the present study should be regarded as a negative trial with respect to the primary wrinkle outcomes. However, several aspects of the secondary endpoint profile, particularly the modest improvement in the global elasticity index R2, together with the favourable safety profile, provide insights into the potential of this postbiotic as a skin health ingredient.

Both groups showed significant intra-group improvements in wrinkle severity, hydration, TEWL, and elasticity over 12 weeks, which is common in dermatologic supplementation studies; participation in a trial can help ensure consistent skincare, greater sun protection, and better daily routines, whereas seasonal or environmental factors during the April-November study period may favour barrier recovery [16,18]. The comparable improvements in wrinkle measures and barrier-related parameters in both groups indicate a relatively strong placebo or background effect, which may have reduced the ability to detect product-specific benefits at the primary endpoints.

However, the modest but statistically significant difference observed in the global elasticity index R2 presents an exploratory finding. R2 represents the overall elasticity (Ua/Uf) and is widely used as an integrated measure of the mechanical performance of the skin. In the present trial, R2 increased in both groups; however, the magnitude of improvement was slightly greater in the ID-ACT 3302 group, reflecting a significant inter-group difference at week 12. At week 12 (PP set; ID-ACT 3302 *n =* 43; placebo *n =* 46), the baseline-adjusted change in the global elasticity index R2 (Ua/Uf) was greater in the ID-ACT 3302 group than in placebo (LS-mean change 0.04 ± 0.01 [95% CI 0.01–0.06] vs. −0.00 ± 0.01 [−0.03–0.02]; ANCOVA *p* = 0.0334). However, the standardized effect size based on raw change scores was small (Cohen’s d ≈ 0.10), consistent with the direction expected from the preclinical profile of ID-ACT 3302, which includes the upregulation of barrier proteins and modulation of extracellular matrix-related pathways [12,13]. Similar tendencies, including modest improvements in elasticity parameters after several months of supplementation, have been reported for other probiotic and postbiotic preparations [8,15,17].

In contrast, R5 and R7, and global ratings improved to a similar extent in both groups. This potentially reflects differential sensitivity: as a global measure, R2 captures subtle changes that are not manually perceived within 12 weeks, especially in a relatively healthy population with mild-to-moderate photoaging. Our data indicate that ID-ACT 3302 may contribute to small instrument-detectable improvements in global elasticity, instead of dramatic, clinically obvious changes within a short time frame.

The lack of significant inter-group differences in hydration and TEWL outcomes should be further considered. Both ID-ACT 3302 and placebo improved hydration and reduced facial TEWL in this study. In adults with largely intact baseline barrier function, non-specific factors, such as regular clinic visits, standardized measurement conditions, provision of moisturizers, and generic capsule intake, can promote gradual barrier optimisation. Such background improvements may lead to underestimation of ID-ACT 3302-induced effects, which can be clarified using a longer follow-up period, more stressed populations, or more sensitive biomarker endpoints. Furthermore, a standard moisturizer was provided to participants in both groups during the trial as part of the study procedures. Because topical moisturizers can improve skin hydration and barrier-related parameters and may also influence the clinical appearance of fine lines, this co-intervention could have contributed to the within-group improvements observed, particularly in the placebo group. Consequently, moisturizer use may have reduced between-group contrasts and our ability to detect a placebo-adjusted effect on the prespecified primary endpoint. These design elements including seasonal or environmental factors likely increased background improvement and reduced assay sensitivity for detecting placebo-adjusted effects on wrinkle outcomes. Improvements in hydration and barrier function can translate into short-term changes in the visual appearance of fine lines, which may narrow between-group differences even when a modest product-specific effect exists. Therefore, the null primary result should be interpreted in the context of co-intervention and environmental effects that likely influenced both groups similarly.

Additionally, multiple comparison-related issues should be considered. The present trial evaluated numerous endpoints: wrinkle parameters at different time points and facial sites, hydration, TEWL, multiple elasticity indices, and global ratings; however, no formal adjustment for multiplicity was applied to the secondary endpoints. Hence, the significant inter-group variation in R2 may be an accidental outcome. Therefore, we consider this finding as hypothesis-generating, instead of confirmatory, and recommend its replication in future trials with hierarchical testing strategies or appropriate multiplicity control.

Despite these limitations, this study revealed the clinical profile of ID-ACT 3302, which is reasonably consistent with the mechanistic data on ID-ACT 3302 and related strains. In vitro and in vivo studies have indicated that heat-killed *L. acidophilus* IDCC 3302 attenuates UV-induced oxidative and inflammatory responses, modulates matrix metalloproteinase activity, and upregulates the expression of filaggrin and involucrin [12,13,14]. These mechanisms are directly related to skin barrier integrity and elasticity. Other postbiotic and probiotic trials have reported similar effects, with modest but favourable changes in elasticity and barrier function after several months of supplementation [14,15,16,17,18]. In this context, our findings present a consistent early clinical signal, instead of definitive proof of a large cosmetic effect.

Furthermore, this trial revealed the safety and tolerability profiles of ID-ACT 3302. Over 12 weeks, ID-ACT 3302 treatment was associated with a few mild AEs, which were considered unrelated to the study product. We detected no serious AEs, clinically meaningful changes in laboratory parameters, vital signs, or body weight. As postbiotics are often selected owing to their stability and safety advantages over live strains, these data justify the use of ID-ACT 3302 as a safe daily ingredient in functional food formulations for improved skin health [25].

Therefore, the present study has certain limitations. First, as noted above, the primary wrinkle endpoints were negative, and the observed benefit in R2 emerged only among several secondary endpoints without adjustment for multiplicity. Second, the study duration was potentially insufficient to observe the full magnitude of the product effects, particularly for slowly varying endpoints, such as deep wrinkle structures and dermal remodelling. Third, the study population consisted of relatively healthy Korean adults with mild-to-moderate photoaging, which limits the generalisability of the findings to other ethnicities, age groups, or individuals with more pronounced barrier impairment. Unmeasured lifestyle factors, including detailed dietary patterns, stress levels, and day-to-day skincare variations, may have influenced the outcomes despite efforts to standardize key aspects. In addition, although conventional wrinkles and biophysical endpoints are widely used and accepted, they may be insufficiently sensitive to early molecular or structural changes; the incorporation of biomarkers related to collagen turnover, oxidative stress, or microbiome shifts could provide deeper insights.

Therefore, future studies on ID-ACT 3302 should consider longer intervention periods, enrollment of populations with a more compromised skin barrier function or more advanced photoaging, and integration of biomarker-based endpoints to better characterize the host–microbe–skin axis in vivo. Stratified analyses may help identify subgroups with potentially greater benefits, such as individuals with higher baseline TEWL or lower baseline elasticity. Comparative studies of live probiotics or other postbiotics could comparatively elucidate the advantages of heat-killed preparations in terms of efficacy and formulation [18,26,27]. Prospective trials with prespecified hierarchical testing of primary and key secondary endpoints and appropriate adjustment for multiplicity are crucial for confirming the reproducibility of the R2 signal observed in this study.

Several limitations of this study should be considered. The 12-week duration and inclusion of relatively healthy adults with mild-to-moderate photoaging may have limited the detection of small and slowly developing changes, particularly in deeper wrinkle morphology. Background effects and co-interventions may also have improved outcomes in both groups, diminishing between-group contrasts. Additionally, participant-reported self-assessment scores are inherently subjective and may be influenced by expectancy effects or differential reporting; therefore, they should be interpreted as supportive outcomes alongside the objective instrument-based measures. In addition, multiple secondary outcomes were assessed without formal multiplicity adjustment; therefore, the verified difference observed in R2 should be understood as hypothesis-generating.

In summary, ID-ACT 3302 did not demonstrate superiority over placebo for the prespecified primary wrinkle endpoints, despite improvements observed in both groups. A small between-group difference in R2 was observed; however, this isolated signal was not supported by other elasticity indices (R5 and R7) and secondary endpoints were not multiplicity-adjusted. Therefore, the R2 result should be considered exploratory and warrants confirmation in future trials with prespecified hierarchical testing and adequate power.

## 5. Conclusions

In this 12-week randomized, double-blind, placebo-controlled trial, ID-ACT 3302 was safe and well tolerated; however, it did not demonstrate a placebo-adjusted benefit for the prespecified primary wrinkle outcomes. A small between-group difference was observed in the Cutometer overall elasticity index (R2), but given the lack of multiplicity adjustment for secondary endpoints and the modest effect size (and the absence of consistent signals in related elasticity indices), this finding should be interpreted as exploratory. Longer-term, adequately powered studies with prespecified hierarchical testing are needed to confirm whether ID-ACT 3302 confers reproducible benefits on skin elasticity.

## Figures and Tables

**Table 1:** Baseline characteristics.

Characteristic	ID-ACT 3302 (*n* = 43)	Placebo (*n* = 46)	*p*-Value
Age, years	46.56±6.79	46.83±6.53	0.85 ^(1)^
Male, N (%)	6 (13.95%)	3 (6.52%)	0.3049 ^(2)^
Female, N (%)	37 (86.05%)	43 (93.48%)	
Current smoker, N (%)	0	0	-
Alcohol intake, N (%)	15 (34.88%)	11 (23.91%)	0.2554 ^(3)^

Data are presented as mean ± SD or N (%). SD, standard deviation; N, number of participants; ID-ACT 3302, *Lactobacillus acidophilus* IDCC 3302. ^(1)^ *p*-value by Two-sample *t*-test; ^(2)^ *p*-value by Fisher’s exact test; ^(3)^ *p*-value by Chi-square test.

**Table 2:** Week 12 primary wrinkle outcomes (YANUS^®^, PRIMOS^lite^).

Parameter	Group	Change from Baseline (Mean ± SD)	*p*-Within ^(1)^	*p*-Between ^(2)^	*p*-Value Adjusted ^(3)^
YANUS^®^ wrinkle grade (left crow’s foot)	ID-ACT 3302	−0.37 ± 0.62	0.0003	0.9925	0.7524
YANUS^®^ wrinkle grade (left crow’s foot)	Placebo	−0.41 ± 0.54	<0.0001		
YANUS^®^ wrinkle grade (right crow’s foot)	ID-ACT 3302	−0.40 ± 0.58	<0.0001	0.6357	0.6267
YANUS^®^ wrinkle grade (right crow’s foot)	Placebo	−0.46 ± 0.55	<0.0001		
YANUS^®^ wrinkle grade (mean of L/R)	ID-ACT 3302	−0.38 ± 0.50	<0.0001	0.9067	0.6296
YANUS^®^ wrinkle grade (mean of L/R)	Placebo	−0.43 ± 0.47	<0.0001		
PRIMOS^lite^ Ra, μm (mean of L/R)	ID-ACT 3302	−0.07 ± 2.46	0.8581	0.3954	0.2254
PRIMOS^lite^ Ra, μm (mean of L/R)	Placebo	−0.67 ± 1.66	0.0092		
PRIMOS^lite^ Rz, μm (mean of L/R)	ID-ACT 3302	0.70 ± 11.05	0.6816	0.3864	0.1688
PRIMOS^lite^ Rz, μm (mean of L/R)	Placebo	−2.28 ± 7.13	0.0356		
Eye wrinkle volume, mm3 (mean of L/R)	ID-ACT 3302	−0.10 ± 0.62	0.2995	0.4427	0.4027
Eye wrinkle volume, mm3 (mean of L/R)	Placebo	−0.19 ± 0.59	0.0369		

Data are presented as mean ± SD. SD, standard deviation; ID-ACT 3302, *Lactobacillus acidophilus* IDCC 3302; L/R, left/right. ^(1)^ Compared within group; *p*-value by Paired *t*-test; ^(2)^ Compared between groups; *p*-value by Wilcoxon rank sum test; ^(3)^ Compared between groups; *p*-value by ANCOVA adjusted by baseline.

**Table 3:** Week 6 wrinkle outcomes.

Parameter	Group	Change from Baseline (Mean ± SD)	*p*-Within ^(1)^	*p*-Between ^(2)^	*p*-Value Adjusted ^(3)^
YANUS^®^ wrinkle grade (mean of L/R)	ID-ACT 3302	−0.17 ± 0.41	0.0074	0.9119	0.9810
YANUS^®^ wrinkle grade (mean of L/R)	Placebo	−0.18 ± 0.40	0.0030		
PRIMOS^lite^ Ra, μm (mean of L/R)	ID-ACT 3302	0.21 ± 2.24	0.5368	0.0601 ^(4)^	0.0850
PRIMOS^lite^ Ra, μm (mean of L/R)	Placebo	−0.65 ± 2.04	0.0355		
Eye wrinkle volume, mm3 (mean of L/R)	ID-ACT 3302	−0.02 ± 0.54	0.8435	0.3557	0.7115
Eye wrinkle volume, mm3 (mean of L/R)	Placebo	−0.04 ± 0.65	0.6864		

Data are presented as mean ± SD. SD, standard deviation; ID-ACT 3302, *Lactobacillus acidophilus* IDCC 3302; L/R, left/right. ^(1)^ Compared within group; *p*-value by Paired *t*-test; ^(2)^ Compared between groups; *p*-value by Wilcoxon rank sum test; ^(3)^ Compared between groups; *p*-value by ANCOVA adjusted by baseline; ^(4)^ Compared between groups; *p*-value by Two sample *t*-test.

**Table 4:** Skin hydration and TEWL.

Parameter	Time Point	Group	Change from Baseline (Mean ± SD)	*p*-Within ^(1)^	*p*-Between ^(2)^	*p*-Value Adjusted ^(3)^
Facial hydration, AU (mean of L/R facial sites)	Week 6	ID-ACT 3302	14.68 ± 18.67	<0.0001	0.7520 ^(4)^	0.6221
Facial hydration, AU (mean of L/R facial sites)	Week 6	Placebo	15.91 ± 17.89	<0.0001		
Facial hydration, AU (mean of L/R facial sites)	Week 12	ID-ACT 3302	33.16 ± 24.94	<0.0001	0.6815 ^(4)^	0.8308
Facial hydration, AU (mean of L/R facial sites)	Week 12	Placebo	31.28 ± 16.96	<0.0001		
Forearm hydration, AU (mean of L/R volar sites)	Week 6	ID-ACT 3302	5.77 ± 16.64	0.0283	0.9183	0.5278
Forearm hydration, AU (mean of L/R volar sites)	Week 6	Placebo	5.71 ± 14.23	0.0092		
Forearm hydration, AU (mean of L/R volar sites)	Week 12	ID-ACT 3302	7.95 ± 13.65	0.0004	0.4467 ^(4)^	0.0693
Forearm hydration, AU (mean of L/R volar sites)	Week 12	Placebo	5.66 ± 14.54	0.0113		
Facial TEWL, g/h·m^2^ (mean of L/R facial sites)	Week 6	ID-ACT 3302	−1.41 ± 4.41	0.0426	0.0900	0.0870
Facial TEWL, g/h·m^2^ (mean of L/R facial sites)	Week 6	Placebo	0.79 ± 7.60	0.4821		
Facial TEWL, g/h·m^2^ (mean of L/R facial sites)	Week 12	ID-ACT 3302	−2.93 ± 4.58	0.0001	0.8054	0.7084
Facial TEWL, g/h·m^2^ (mean of L/R facial sites)	Week 12	Placebo	−2.57 ± 5.04	0.0012		
Forearm TEWL, g/h·m^2^ (mean of L/R volar sites)	Week 6	ID-ACT 3302	3.60 ± 9.63	0.0185	0.6136	0.6109
Forearm TEWL, g/h·m^2^ (mean of L/R volar sites)	Week 6	Placebo	2.83 ± 10.23	0.0667		
Forearm TEWL, g/h·m^2^ (mean of L/R volar sites)	Week 12	ID-ACT 3302	−0.17 ± 7.01	0.8771	0.5710	0.8581
Forearm TEWL, g/h·m^2^ (mean of L/R volar sites)	Week 12	Placebo	0.39 ± 8.02	0.7449		

Data are presented as mean ± SD. SD, standard deviation; TEWL, transepidermal water loss; ID-ACT 3302, *Lactobacillus acidophilus* IDCC 3302; L/R, left/right; AU, arbitrary units; volar site, volar forearm sites. ^(1)^ Compared within group; *p*-value by Paired *t*-test; ^(2)^ Compared between groups; *p*-value by Wilcoxon rank sum test; ^(3)^ Compared between groups; *p*-value by ANCOVA adjusted by baseline; ^(4)^ Compared between groups; *p*-value by Two sample *t*-test.

**Table 5:** Skin elasticity (R2, R5, R7) and global assessment scores.

Parameter	Time Point	Group	Change from Baseline (Mean ± SD)	*p*-Within ^(1)^	*p*-Between	*p*-Value Adjusted ^(3)^
R2 (Ua/Uf, overall elasticity, mean L/R)	Week 12	ID-ACT 3302	0.02 **± 0.12**	0.2796	0.7509 ^(4)^	0.0334
R2 (Ua/Uf, overall elasticity, mean L/R)	Week 12	Placebo	0.01 ± 0.08	0.3224		
R5 (Ur/Ue, immediate elasticity, mean L/R)	Week 12	ID-ACT 3302	0.08 ± 0.08	<0.0001	0.5943 ^(4)^	0.1413
R5 (Ur/Ue, immediate elasticity, mean L/R)	Week 12	Placebo	0.07 ± 0.10	<0.0001		
R7 (Ur/Uf, elasticity ratio, mean L/R)	Week 12	ID-ACT 3302	0.04 ± 0.05	<0.0001	0.7499 ^(4)^	0.2346
R7 (Ur/Uf, elasticity ratio, mean L/R)	Week 12	Placebo	0.03 ± 0.06	0.0005		
Investigator’s global assessment score	Week 12	ID-ACT 3302	2.14 ± 0.68	-	0.1960 ^(2)^	-
Investigator’s global assessment score	Week 12	Placebo	2.35 ± 0.77	-		
Participant’s global assessment score	Week 12	ID-ACT 3302	2.30 ± 0.64	-	0.7259 ^(2)^	-
Participant’s global assessment score	Week 12	Placebo	2.33 ± 0.63	-		

Data are presented as mean ± SD. SD, standard deviation; ID-ACT 3302, *Lactobacillus acidophilus* IDCC 3302; L/R, left/right; Ua, total retraction (mm); Ue, immediate distention (mm); Uf, total elongation (mm); Ur, Immediate retraction (mm). ^(1)^ Compared within group; *p*-value by Paired *t*-test; ^(2)^ Compared between groups; *p*-value by Wilcoxon rank sum test; ^(3)^ Compared between groups; *p*-value by ANCOVA adjusted by baseline; ^(4)^ Compared between groups; *p*-value by Two sample *t*-test.

**Table 6:** Safety summary (AEs, labs, BP).

Parameter	ID-ACT 3302	Placebo	*p*-Value
Participants with ≥1 adverse event, n (%)	1 (2)	3 (6)	0.6173
Serious adverse events, n	0	0	-
Study discontinuations due to AEs, n	0	0	-
AEs related to study product, n	0	0	-
Change in hemoglobin, g/dL	−0.15 ± 0.60	−0.26 ± 0.54	0.3297
Change in AST, IU/L	−1.96 ± 6.22	−0.44 ± 6.68	0.2418
Change in ALT, IU/L	−2.22 ± 9.32	1.62 ± 11.05	0.0633
Change in creatinine, mg/dL	−0.01 ± 0.06	−0.02 ± 0.08	0.4534
Abnormal urinalysis, n (%)	0	0	-
Change in systolic BP, mmHg	−0.38 ± 7.03	0.40 ± 7.43	0.5909
Change in diastolic BP, mmHg	−2.02 ± 6.18	−0.12 ± 6.64	0.1416
Change in pulse rate, bpm	1.14 ± 8.29	2.10 ± 8.07	0.5587
Change in body weight, kg	0.17 ± 1.20	0.12 ± 0.66	0.7969

## Data Availability

The data presented in this study are available on request from the corresponding author due to reasons of sensitivity.
